# Risk factors of efficacy for patients receiving surgical treatment following terrible triad of the elbow joint: A comparative study

**DOI:** 10.1097/MD.0000000000013836

**Published:** 2019-01-04

**Authors:** Hong-Wei Chen, Shu-Ming Huang

**Affiliations:** a Department of Orthopedic Surgery, Yiwu Central Hospital, The Affiliated Yiwu Hospital of Wenzhou Medical University, Yiwu; b Department of Orthopedics, Lishui Municipal Central Hospital, Lishui Hospital of Zhejiang University, The 5th Affiliated Hospital of Wenzhou Medical University, Lishui, China.

**Keywords:** comparative study, efficacy, factors, surgical treatment, terrible triad of elbow joint

## Abstract

**Background:**

This study aims to explore the efficacy of surgical and conservative treatment for elbow joint terrible triad, and evaluate related factors affecting surgical treatment efficacy.

**Methods:**

Patients with terrible triad of elbow joint (n = 165) were selected, among which 79 cases underwent conservative treatment (the control group) and 86 cases underwent surgical treatment (the experimental group). The range of flexion and extension, range of rotation and Mayo elbow performance score were recorded. In the experimental group, postoperation, according to the Mayo elbow performance score, patients were assigned into the effective group (72 cases) and ineffective group (14 cases). All patients were followed up regularly for 6 to 24 months. X-ray and computed tomography examination were used to examine anterior and posterior elbow joints preoperatively and postoperatively and the degree of arm rotation.

**Results:**

The range of flexion and extension, range of rotation and Mayo elbow performance score were found to be significantly higher in the experimental group after treatment compared to the experimental group before treatment and in the control group after treatment. Seven days after treatment, compared with the control group, the expressions of interleukin (IL)-6, C-reactive protein, IL-8, and tumor necrosis factor-α in serum decreased, and returned to almost near normal levels in the experimental group. Age, mean operative time, and postoperative immobilization time were significantly different between the effective and ineffective groups. The incidence of joint stiffness, heterotopic ossification, and ulnar nerve symptoms in the effective group were lower than those in the ineffective group. The postoperative immobilization time served a protective factor for the efficacy of surgical treatment of elbow joint terrible triad, while age served as a risk factor.

**Conclusion:**

The results indicated that surgical treatment regimens for elbow joint terrible triad exhibited better efficacy than conservative treatment regimens, and lower age and longer postoperative immobilization time serve as protective factors for surgical treatment efficacy.

## 1 Introduction

The elbow is one of the most commonly dislocated joint with an annual incidence of about 6 to 8 cases per 100,000 individuals.^[1]^ Furthermore, 49% of these dislocations are elbow fracture dislocations, which often lead to long-term loss of function and chronic stiffness or instability.^[2]^ One particular type of elbow fracture dislocation is terrible triad of elbow joint.^[3]^ Terrible triad of elbow, put forward by Hotchkiss in 1996, is characterized by elbow dislocation (posterolateral dislocation or LCL injury), radial head or neck fractures, and coronoid process fractures.^[4]^ This injury is known to occur when the forearm is in supination, the elbow joint is extended and/or abducted, and great force is exerted in the axial direction.^[5]^ However, it remains to be a disease that is difficult to cure and has poor prognoses owing to persistent elbow instability and other complications such as malunion, arthrofibrosis, heterotopic ossification, and ulnar neuropathy.^[6,7]^ Therefore, it is essential to further increase the understanding of the injury mechanism, relevant anatomy, and factors related to elbow stability to effectively treat this.^[8]^

The current treatment regimens for terrible triad of elbow joint differ on the basis of bone pathology and ligament injuries, including closed reduction, nonsurgical treatment and surgical treatment by the use of external fixation, open reduction and internal fixation, excision arthroplasty of radial head, or radial head replacement.^[9,10]^ Previous studies have revealed that conservative treatment regimens including manipulative reduction and plaster immobilization often achieve unsatisfactory results owing to redislocation of the ulno-humeral joint, and thereby now consider the surgical approach as the optimal treatment option.^[9]^ Surgical treatment includes recovering radiocapitellar contact (through fixation or replacement of radial head), reattaching the origin of the lateral collateral ligament to the lateral epicondyle and conventional or selective fixation of the coronoid fracture.^[11]^ Operative treatment is aimed at restoring the anatomic structures, repairing the collateral ligament and articular capsule.^[12]^ A previous study found that a combined posterior lateral and anteromedial approach achieved good results in the treatment of terrible triad of the elbow joint with increased fracture healing rate, improved recovery of elbow functions, and decreased complications.^[13]^ Moreover, restoration of the radial head, coronoid, medial, and lateral collateral ligaments through operative treatment has been known to help recover elbow stability, allow early postoperative motion and promote the recovery of elbow functions.^[14]^ Therefore, the present study aims to compare conventional treatment with surgical treatment for terrible triad of the elbow joint, and analyze the related factors affecting surgical efficacy.

## 2 Materials and methods

### 2.1 Study subjects

A total of 165 patients diagnosed with terrible triad of elbow joint (including 91 males, and 74 females; aged between 30 and 55 years) at the Yiwu Central Hospital and Lishui Municipal Central Hospital from March 2001 to January 2015 were included in the present study. All included patients underwent surgical treatment ∼4 days after injury (from the same day of the surgery to the 9th day after surgery). All included patients presented with unilateral fractures, of which 112 cases were in the left limb, and 53 cases in the right limb. Among the 165 patients, 99 cases were attributed to car accidents, and 66 cases due to fall-type injuries. Fractures of the coronoid process of ulna were regarded as type I (avulsion fracture of the coronoid process of ulna) according to the Regan-Morrey classification.^[15]^ The experimental group was established using the 86 cases receiving surgical treatment, and the remaining 79 cases receiving conservative treatment were regarded as the control group. All patients underwent frontal and lateral X-ray and computed tomography (CT) examination of the elbow joints. In the experimental group, 12 months after the operation, the curative effect was evaluated according to the Mayo elbow performance score. Inclusion criteria were as follows: all patients were inpatients and could physically tolerate surgical treatment; all patients were over 18 years old and presented with fresh fractures; all patients exhibited active elbow joint functioning prior to injury; all patients conformed to the diagnosis of terrible triad of elbow joint and indications of surgical treatment. Exclusion criteria were as follows: patients not in conformity with the inclusion criteria; patients presenting with nerve injuries on the affected side, or history of hemiplegia or myasthenia gravis which may affect postoperative joint function exercise; patients with old fractures; patients with serious primary diseases, such as cardiovascular disease and diabetes, mental diseases, or with poor overall condition that did not allow for operations. The present study was approved by the Ethics committee of the Yiwu Central Hospital and Lishui Municipal Central Hospital, and signed informed consents were obtained from all patients and guardians.

### 2.2 Preoperative preparation

Various parameters including the activity and strength of elbow joint flexion, extension, and rotation as well as symptoms of nerve injury were observed and noted. Routine X-ray examination, CT plain scanning, and 3-dimensional reconstruction examination were performed prior to the operation to check planeness of articular surfaces, degree of joint space stenosis, and to determine the severity of heterotopic ossification, respectively.

### 2.3 Surgical methods

Individual specific conditions and family economic situations directed whether the patients chose to proceed with conservative treatment or surgical treatment. In the experimental group, the patients were anesthetized using the brachial plexus block technique or tracheal intubation in the supine position. A pneumatic tourniquet was used in the affected limb, and the body surface was marked and positioned using lines. All surgical procedures were performed according to McKee principles of surgical treatment for terrible triad of elbow joint.^[16]^ An incision was made employing the elbow anteromedial approach, initiating from epitrochlea, down crossing the elbow stripes and extending to the upper end of the ulna. After layer-by-layer incisions, the flexor muscles were separated longitudinally. The anteromedial side of the elbow joint was observed, and the medial collateral ligament (deep layer) and medial joint capsule were exposed. Next, the articular capsule was incised until the ulna coronal fracture was exposed. Then, the lacerated medial joint capsule and collateral ligament were repaired, and the incision was closed layer-by-layer. An oblique incision of posterolateral elbow was made, and the articular capsule was cut between the elbow muscle and extensor carpi ulnaris. Subsequently, the radius fracture was restored. Next, the anterior joint capsule, radial head fracture, lateral collateral ligament, and origin of common extensor tendon were repaired. After the operation, the patients underwent X-ray examinations every week for a duration of 1 month to ensure that there was no immediate subluxation or redislocation. All patients in the control group underwent conservative treatment, and the procedures of conservative treatment were as follows: in the acute stage, analgesic drugs were administered to the patient when awake, and then treated with manual closed reduction. After performing reduction, the elbow joint was flexed to an angle of 90°, and the neutral position of the forearm was fixed using a plaster slab for a duration of 3 to 7 days. X-ray and physical examinations were performed to determine the degree of ulna coronoid process and radial head fracture. If the results met the expected indications for conservative treatment, the patient was allowed to initiate early functional exercises within a steady radian range 10 days after reduction.

### 2.4 Postoperative management

After the operation, the elbow joint was flexed to an angle of 90°, and fixed using a plaster slab in the neutral position of the forearm. Subsequently, the routine indwelling drainage tube was extracted 24 to 48 hours after surgery. Two days after performing the surgery, various functional exercises, including active elbow flexion and stretching and passive forearm rotation, were initiated under the supervision of a doctor. It was noted that the joint range of motion gradually increased with every progressing week. Indomethacin (Shanghai Shu Can Industrial Co, Ltd, Shanghai, China) was administrated postoperatively to patients in a routine manner for 3 weeks to prevent heterotopic ossification, and parecoxib (Wuhan Dahua Weiye Pharmaceutical Chemical Co, Ltd, Wuhan, China) for a duration of 6 weeks to relieve pain.

### 2.5 Trauma index detection

Venous blood samples (3 mL) were obtained from fasted patients in the experimental and control groups using the median cubital vein prior to the treatment as well as on the 1st and 7th day after treatment. The extracted blood samples were added to a procoagulant test tube, centrifuged, and placed in a refrigerator at −20°C for further experimentation. Enzyme-linked immunosorbent assay (ELISA; Beijing North Biotechnology Research Institute, Beijing, China) was employed to detect numerous indexes including C-reactive protein (CRP), interleukin (IL)-6, IL-8, and tumor necrosis factor-α (TNF-α) of serum, and all operations were carried out in strict accordance with the manufacturer's instructions.

### 2.6 X-ray examination

A digital X-ray camera, FSK302-1A type 500 mA X machine (Beijing Wandong Medical Equipment Co, Ltd, Beijing, China) was employed to perform X-ray examinations in all patients, and the voltage was set to 58 to 70 kV, and the current was set to 8 to 15 mA.

### 2.7 CT examination

The GE Prospeed-AI spiral CT machine (D3162T; General Electric Company; Boston, USA) was employed to perform CT examinations on all patients, and the scanning range was determined by scanning location image. The scanning layer thickness was 2 to 5 mm, the pitch of screw was 2 to 5 mm, and the layer thickness was 1 to 2 mm. After performing the scans, the volume data were transmitted to a workstation for further processing. All images were evaluated by 2 experienced professional physicians using the double-blind method, and consensus was achieved following proper discussion of conflicting opinions.

### 2.8 Follow-up and efficacy evaluation

In the present study, 165 patients were followed up for a ranging duration of 6 to 24 months (the follow-up rate was 99.6% in the first 6 months), with an average follow-up of 13.8 months. The follow-up contents were as follows:

1. frontal and lateral X-ray films of the elbow joint;
2. elbow flexion, extension, degree of flexion and extension, pronation, supination, and elbow rotation range;
3. treatment efficacy; efficacy was evaluated 12 months after operation using the Mayo elbow performance score system, including pain (45 points), flexion and extension (20 points), joint stability (10 points), and daily activity function (25 points); the patients scoring ≥90 points were regarded as with excellent efficacy, scoring 75 to 89 points as with good efficacy, 60 to 74 points as with moderate efficacy, and <60 points as with poor efficacy; the patients with excellent or good efficacy were grouped as the effective group, and those with moderate or poor efficacy were grouped as ineffective group;
4. postoperative complications; incidence of complications and immobilization time of the affected limb were recorded.

### 2.9 Statistical analysis

Statistical analyses were performed using the SPSS 21.0 software version (IBM Corp, Armonk, NY). Measurement data were expressed as mean ± standard deviation. Comparisons between 2 groups were analyzed using the *t* test, and multi-group comparisons were performed using analysis of variance. Enumeration data were expressed as percentage or rate. In addition, comparisons between groups were validated using the Chi-squared test. Related factors and surgical efficacy were analyzed by logistic multiple regression analysis. *P* < .05 was considered to be statistically significant.

## 3 Results

### 3.1 Baseline characteristics between the experimental and control groups

Compared with the control group, the indexes including age, gender, cause of injury, injury site, body mass index (BMI) value, hospitalization time after treatment were not found to be significantly different from the results of the experimental group (all *P* > .05) (Table 1).

**Table 1:**
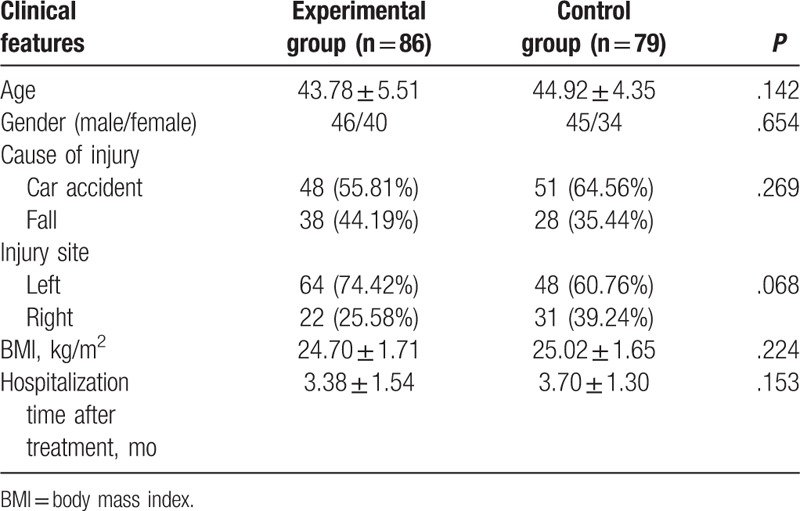
Baseline characteristics between the control and experimental groups.

### 3.2 X-ray films in the experimental and control groups

**Figure 1:**
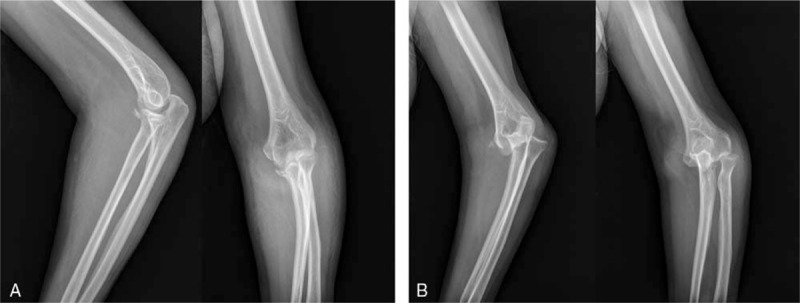
Fracture site of elbow joint in the experimental and control groups. (A) Postoperative frontal and lateral X-ray of elbow joint in the experimental group. (B) Postoperative frontal and lateral X-ray of elbow joint in the control group.

All fractures were observed to be healed in the experimental group (Fig. 1A). As demonstrated by the X-ray film, the fracture site was found to be fixed, reduction was good and there was no evidence of elbow joint dislocation. In addition, the film revealed significantly improved fracture situations. However, the control group exhibited poor fracture healing (Fig. 1B).

### 3.3 Elbow functioning before and after treatment in the experimental and control groups

Prior to the treatment, no significant differences were found in the range of flexion and extension, the range of rotation or the Mayo elbow performance scores between the experimental group and control group (all *P* > .05). However, multiple parameters including range of flexion and extension, range of rotation, and Mayo elbow performance score were found to be significantly improved in the experimental group after treatment compared to those in the experimental group before treatment and those in the control group after treatment (all *P* < .05) (Table 2).

**Table 2:**
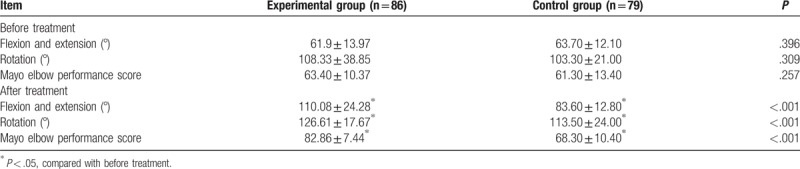
Elbow joint function evaluation before and after treatment.

### 3.4 Expression of CRP, IL-6, IL-8, and TNF-α in the experimental and control groups

The ELISA was employed to detect the serum expressions of CRP, IL-6, IL-8, and TNF-α at various time points (Table 3). All indexes were found to be higher on the 1st day after treatment compared to prior to the treatment, while exhibiting a decrease 7 days after treatment. No significant differences were found in the expression of IL-6 and TNF-α between the samples procured before the treatment and after 7 days after treatment (both *P* > .05); however, the expressions of IL-8 and CRP were found to be significantly different (both *P* < .05). On the 1st day after administering treatment, the expressions of CRP, IL-6, IL-8, and TNF-α were found to evidently lower in the experimental group in comparison with the control group (all *P* < .05). Furthermore, the experimental group presented with significantly decreased serum expressions of CRP, IL-6, IL-8, and TNF-α compared to the control group after 7 days of administering treatment (all *P* < .05).

**Table 3:**
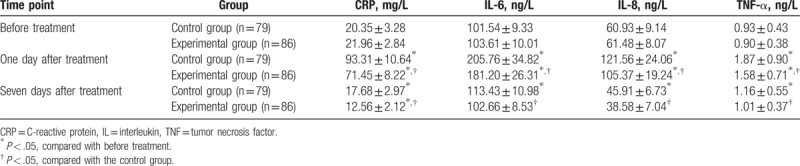
CRP, IL-6, IL-8, and TNF-α expression in serum in the experimental and control groups.

### 3.5 Clinical features in the effective and ineffective groups

As shown in Table 4, there were 72 patients in the effective group and 14 patients in the ineffective group. Compared with the ineffective group, the indexes including gender, cause of injury, location of injury, and BMI value were found to be not significantly different from those in the effective group (all *P* > .05). However, the effective group presented with lower average age, shorter mean operation time, and longer postoperative immobilization time in comparison with the ineffective group (all *P* < .05).

**Table 4:**
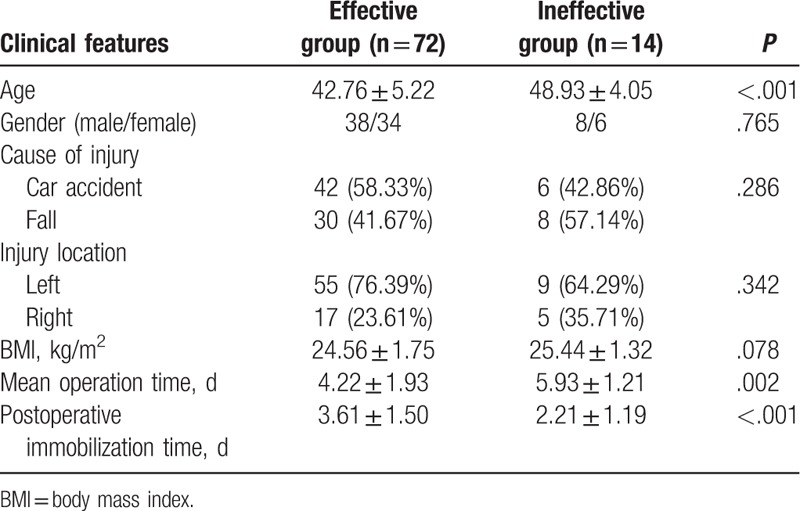
Comparison of clinical features between the effective group and the ineffective group.

### 3.6 Complication incidence in the effective and ineffective groups after operation

Postoperative complications in the effective and ineffective groups were as follows: the incidence of elbow instability, healing difficulty, wound infection, and myositis ossificans were found not to be significantly different between the effective and ineffective groups (all *P* > .05). However, the effective group presented with significantly lower incidence of joint stiffness, heterotopic ossification in addition to ulnar nerve symptoms than those in the ineffective group (all *P* < .05) (Table 5).

**Table 5:**
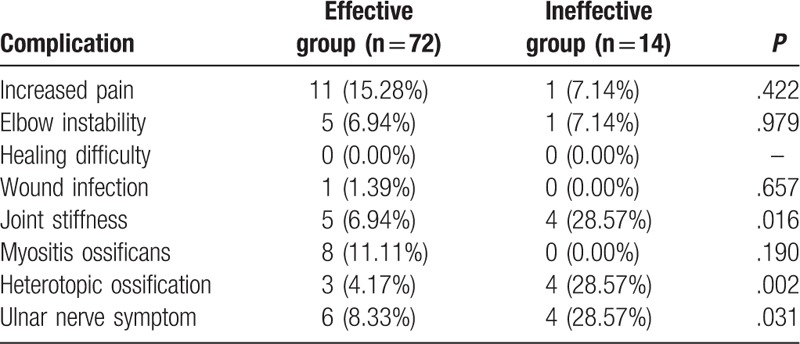
Comparison of postoperative complication incidence between the effective and ineffective groups.

### 3.7 Related factors of treatment efficacy for patients with terrible triad of elbow joint

With the effective and ineffective efficacy serving as dependent variables, a logistic regression analysis was performed using 3 factors, including average age, mean operation time, and postoperative immobilization time. The postoperative immobilization time served as a protective factor of surgical treatment for patients with terrible triad of elbow joint, while age served as a risk factor. However, operation time exerted no significant effects on the surgical treatment for patients with terrible triad of elbow joint (Table 6).

**Table 6:**
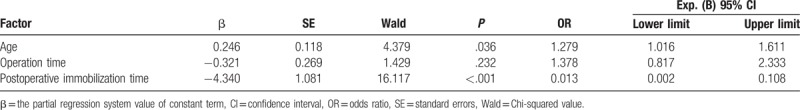
Logistic regression analysis of related factors affecting surgical efficacy.

## 4 Discussion

Terrible triad of elbow is a severe disease presenting with difficult treatment regimens and poor prognoses including frequent redislocations.^[17]^ The present study investigated the efficacy of surgical and conservative treatments for terrible triad of the elbow joint and related factors affecting surgical treatment efficacy. The findings of the present study highlighted that surgical treatments for elbow joint terrible triad are more efficacious compared to conservative treatments, and lower age and longer postoperative immobilization time serve as protective factors of surgical treatment for this disease.

The present study revealed that patients suffering from terrible triad of the elbow joint undergoing surgical treatment exhibited better outcomes than those who received conservative treatment regimens. In addition, our results evidenced that patients undergoing surgical treatment presented with higher range of flexion and extension, range of rotation, and Mayo elbow performance score than those receiving conservative treatment. Furthermore, conservative treatment regimens have been previously reported to bare unsatisfactory results, given that fracture site prone to redislocation.^[18]^ Patients undergoing surgical treatment through a combined posterior lateral and anteromedial approach, however, showed increased fracture healing rate, improved elbow functioning, and decreased incidence of complications.^[13]^ Similarly, another research demonstrated that surgical treatment regimens for terrible triad of elbow joint, such as radial head arthroplasty, repairing the coronoid or the joint capsule, and repairing the lateral ligament complex of the elbow, accomplished good results in majority patients, which was independent from treatment for radial head fractures.^[19]^ Moreover, it was found that the hinged external fixator technique fared superior results compared to other treatments, because it aided lateral stability of elbows and functioning of the elbow joint in addition to decrease elbow stiffness and heterotopic ossification.^[20]^ Operative treatment contributes to the recovery of elbow stability to allow its early motion while nonoperative treatment needs close clinical and radiographic follow-up to monitor subluxation or fracture displacement in addition to being a selective method as it can only be used in selected patients.^[21]^ In addition, serum levels of IL-6, CRP, IL-8, and TNF-α postoperation were found to be significantly decreased compared with those after conservative treatment. Interestingly, a previous study reported that high serum levels of IL-6 are associated with poor prognoses.^[22]^ Furthermore, another study showed the gradual decrease of serum levels of CRP, IL-6, IL-8, and TNF-α until they reached normal levels after surgical treatment for terrible triad of the elbow joint, which was consistent with our results.^[23]^

Additionally, the present study also demonstrated that lower age and longer postoperative immobilization time serve as protective factors of surgical treatment for terrible triad of elbow joint. Various factors such as bone repair and osteogenic potential are known to decrease with age, exerting a negative influence on fracture repair in elderly people.^[24]^ Similarly, increasing studies have evidenced that bone healing is delayed with increasing age.^[25]^ Another study found that the process of bone formation to bridge a fracture gap after the occurrence of skeletal fractures slows with age, which may be related to alterations in mRNA expressions of specific genes at the bone formation site.^[26]^ Moreover, a previous study revealed that advanced age was regarded as a risk factor for recurrent tearing after rotator cuff repair operation, possibly because elderly patients presented with damaged biomechanical strength as well as histologic organization at the tendon-to-bone junction.^[27]^ Besides, postoperative immobilization has been established as the standard method for rehabilitation of flexor tendon injuries.^[28]^ In addition, postoperative immobilization has been regarded to avoid further damage to the repaired structures and early failure by protecting the repair site from excessive force.^[29]^ Previously, a study further demonstrated that postoperative immobilization could significantly reduce cellularity and apoptosis, which may prevent the stretching of shortened collagenous scaffold, and further improve surgical efficacy following radiofrequency shrinkage.^[30]^ Zhao et al suggested that repaired tendons could undergo immobilization for 10 days with no risk of adhesions, which may be beneficial for the coupled tissue injuries to become tolerant for mobilization later.^[31]^ Moreover, a prolonged course of immobilization and protected weight bearing was usually adopted after surgical repair of ruptured quadriceps tendons.^[32]^

In conclusion, the present study provided evidence indicating that surgical treatments are clearly the superior mode of treatment for terrible triad of elbow joint compared to conservative treatment regimens, and lower age and longer postoperative immobilization time serve as protective factors for terrible triad of elbow joint, and further play positive roles in the efficacy of surgical treatments. However, the limited sample size and follow-up duration serve as limitations to the present study, and further case collection and experiments are warranted in future studies to improve the quality of life of patients suffering from terrible triad of elbow joint.

## Acknowledgment

The authors show sincere appreciation to the reviewers for critical comments on this article.

## Author contributions

**Conceptualization:** Hong-Wei Chen, Shu-Ming Huang.

**Data curation:** Hong-Wei Chen, Shu-Ming Huang.

**Formal analysis:** Hong-Wei Chen, Shu-Ming Huang.

**Funding acquisition:** Hong-Wei Chen, Shu-Ming Huang.

**Investigation:** Hong-Wei Chen, Shu-Ming Huang.

**Methodology:** Hong-Wei Chen.
